# A new initiating system based on [(SiMes)Ru(PPh_3_)(Ind)Cl_2_] combined with azo-bis-isobutyronitrile in the polymerization and copolymerization of styrene and methyl methacrylate

**DOI:** 10.1080/15685551.2016.1231049

**Published:** 2016-10-21

**Authors:** Abdullah M. Al-Majid, Waseem Sharaf Shamsan, Abdel-Basit Mohammed Al-Odayn, Fady Nahra, Taieb Aouak, Steven P. Nolan

**Affiliations:** ^a^ Chemistry Department, College of Science, King Saud University, Riyadh, Saudi Arabia; ^b^ EaStCHEM School of Chemistry, University of St Andrews, St Andrews, United Kingdom

**Keywords:** Bulk polymerization, bulk copolymerization, styrene, methyl methacrylate, ruthenium dicloride-indenylidene/Azo-bis-isobutyronitrile system, structure/microstructure

## Abstract

The homopolymerization and copolymerization of styrene and methyl methacrylate, initiated for the first time by the combination of azo-bis-isobutyronitrile (AIBN) with [(SiMes)Ru(PPh_3_)(Ind)Cl_2_] complex. The reactions were successfully carried out, on a large scale, in presence this complex at 80 °C. It was concluded from the data obtained that the association of AIBN with the ruthenium complex reduces considerably the transfer reactions and leads to the controlled radical polymerization and the well-defined polymers.

## Introduction

In the recent decades, controlled radical polymerization (CRP) technique has attracted much attention, and was used to control important characteristics of vinylic polymers and copolymers mainly molecular weight, molecular distribution and the architectural structure.[1–10] These features are practically impossible to acquire by the traditional radical polymerization method when used alone. Among the most widely used techniques to obtain stereoregular polymers with narrow polydispersity index, are the atom transfer radical polymerization (ATRP) [1] and the reverse atom transfer radical polymerization.[1] A wide range of propagation regulators such as dithioesters, trithiocarbonates, alkoxyamines and xanthates were used to control the different aforementioned properties in combination with organometallic catalysts based on transition metal such as Cu, Fe, Ru, Re, Ni.

The catalytic chain transfer to the monomer and catalytic inhibition were discovered by different researchers [2–6] based on the polymerization of methacrylates using the cobalt-porphyrins system. Wayland et al. [11,12] used the metal complex cobalt-tetramesitylporphyrin in the polymerization of acrylates to achieve homopolymeres and block copolymers. Only a few investigations on the well soluble metal-porphyrin complexes were reported in the literature.[11–15] Notably, those with rhodium, cobalt and aluminum metal centers were used to control the radical polymerization of vinylic monomers. Complexes of Ti(IV) and Zr(IV) with porphyrins were also employed in radical polymerization of vinyl monomers by Islamova et al. [5] It was found from this study that the metal complexes of porphyrins played a crucial role in all stages of polymerization process and in molecular weight characteristics of the resulted polymers. It was also shown that the performance of metal complexes of porphyrins was closely linked to the structure of the polymer obtained.

Ruthenium–indenylidene complexes are mostly known through the literature as very efficient promoters used of the ring-closing metathesis reaction.[16–19] The use of these catalyst systems in atom transfer radical addition (ATRA) is relatively recent. Indeed, Verpoort et al. [20] has employed this technique to polymerize important number of olefins in chloroform and carbon tetrachloride such as acrylates, methacrylates, styrene and some terminal alkenes. According to the latter reports, these reactions could be successfully extended to an ATRP polymerization by controlling the monomer/halide ratio. It was also shown that the addition of *n*-Bu_2_NH to the reaction mixture accelerated the reaction and led to an uncontrollable polymerization. Furthermore, it was suggested that the catalytic activity can be enhanced by the transformation of the Ru complexes in the cationic 14-electron species or exchanging the indenylidene fragment with an ethoxycarbene. Simal et al. [21] employed a series of ruthenium-based catalytic systems to polymerize methylmethacrylic and styrene via ATRP route. They revealed that only [RuCl_2_(p-cymene)(PR_3_)] complexes, bearing both strongly basic and bulky ligands such as P(i-Pr)_3_, P(cyclohexyl)_2_Ph, P(cyclohexyl)_3_, and P(cyclopentyl)_3_), were efficient catalysts for the controlled ATRP of these monomers. De Clercq and Verpoort [22] examined the ATRP of vinyl monomers mediated by a class of ruthenium alkylidene catalyst system containing 1,3-dimesityl-45-dihydroimidazol-2-ylidene (SIMes) combined with a Schiff base as ligand. The role of the solvent, when cationic complexes were used, proved to be crucial for the dynamic and controllability of the metal-catalyzed polymerization. Under biphasic conditions (aqueous/organic), these cationic systems led to the radical polymerization of methyl methacrylate and styrene, in which the resulting polymers had narrow molecular weight distributions. Cationic complexes of ruthenium (II), such as [Ru(o-C_6_H_4-2_-py)(phen)(MeCN)_2_]^+^, bearing different counter ions of ${\text{PF}}_{6}^{-}$ and Cl^−^ were used by Diaz et al. [23] in the radical polymerization of 2-hydroxyethyl methacrylate in protic solvents and acetone, under homogeneous conditions. The exchange of ${\text{PF}}_{6}^{-}$ by Cl^−^ led to an increase in the solubility of the complex in water. Controlled polymerization was only achieved using acetone and methanol. Under the latter conditions, the ruthenium complex containing Cl^−^ as a counter ion, instead of ${\text{PF}}_{6}^{-}$, gave better results.

In this work Azo-bis-isobutyronitrile (AIBN) traditionally used alone as initiator for free radical polymerization combined with ruthenium dichloride–indenyllidene complex, (SiMes)Ru(PPH_3_)IndCl_2_, was prepared for the first time and used as initiator system used as controlled radical homopolymerization and copolymerization of typical vinylic monomers such as methyl methacrylate and styrene. The molecular weight distribution was determined by size exclusion chromatography (SEC) analysis, the structure and microstructure of homopolymers and copolymers (tacticity) were characterized by FTIR and NMR methods. The reactivity ratio of poly(styrene-*co*-methyl methacrylate) and the distribution order of the comonomeric units in the chains were determined by the Mayo–Lewis method. The thermal properties of PMMA, PSt and PSMMA were studied by DSC and TG-DTG methods.

## Experimental

### Materials

Styrene (St) (99.3% purity) and methyl methacrylate (MMA) (99.8 wt% purity) supplied by Sigma Aldrich company were distilled under reduced pressure. AIBN (Aldrich, 98% purity) was purified several times by recrystallization in methanol. The ruthenium complex was synthesized using the literature procedure.[24]

### Synthesis of (SiMes)Ru(PPh_3_)IndCl_2_(M_20_)

In the glovebox, [RuCl_2_(PPh_3_)_2_(Ind)] (M_10_) (1.00 g, 1.13 mmol) and NHC (SIMes, 366 mg, 1.18 mmol) were charged into a Schlenk flask and dissolved in toluene (3 mL). The reaction was taken out of the glovebox, stirred at 40 °C for 3 h under argon. After this time, the mixture was allowed to cool to room temperature and hexane (30 mL) was added to precipitate the product, the suspension was cooled at −40 °C. Filtration and washing with cold methanol (1 × 4 mL) and cold hexane (4 × 10 mL) afforded M_20_ (920 mg, 88%) as a microcrystalline solid.

### Polymerization and copolymerization

To prevent impurities that can be influenced the experimental results, the distillation of monomers and their polymerization were performed simultaneously in the same apparatus in which a known amount of St, MMA was distillated under reduced pressure (10^−4^ mmHg) then directly transferred into a well degassed reactor containing a known amount of initiator system (M_20_/AIBN). All polymerization and copolymerization reactions occurred in bulk under purified gas nitrogen at 80 °C during 2 h. To reduce the experimental errors due to the polymerization conditions such as temperature and weighing, the results of each polymerization and copolymerization were taken from the arithmetic average realized for three experimentations and the experimental conditions are gathered in Table 1.

**Table 1.** Polymerization conditions of styrene, methyl methacrylate and their copolymerization.

Copolymer	St (g)	St (mol. × 10^2^)	MMA (g)	MMA (mol. × 10^2^)	Yield (wt %)
PS	3.64	3.50	–	–	79.38
PSMMA90	3.27	3.14	0.37	0.37	81.25
PSMMA75	2.81	2.70	0.91	0.91	74.48
PSMMA50	1.87	1.80	1.87	1.87	75.55
PSMMA25	0.91	0.88	2.81	2.81	75.18
PSMMA10	0.37	0.36	3.27	3.27	89.03
PMMA	–	–	3.74	3.74	76.75

Notes:

[AIBN] = 2.0 mg (1.22.10^−5^ mol); M_20_ = 11.3 mg (1.22.10^−5^ mol); aging time of AIBN/M_20_ system: 5 min; aging temperature: 80 °C; polymerization temperature: 80 °C; polymerization time: 2 h; M_20_/AIBN = 1.0 (mol mol^−1^).

To determine the reactivity ratio of each comonomer the conversion was limited at a maximum of 10 wt%, corresponding to 20 min. The experimental conditions are shown in Table 2. Poly(styrene)(PSt), poly(methyl methacrylate) (PMMA) and poly(styrene-co-methyl methacrylate) (PSMMA) obtained were purified several times by dissolution in tetrahydrofuran (THF) and reprecipitation in heptane then dried at 60 °C, under vacuum for 24 h. The polymers were separately dissolved at 10 wt% of polymer in THF to form polymeric solutions. The polymer films were prepared by smoothly casting polymeric solutions over a Teflon-plate surface. The plate was then heated at 40 °C for 24 h and dried under vacuum at 50 °C for 24 h. The average thickness of each film was 160 μm.

**Table 2.** Molar fractions of comonomers St (*f*_St_) and MMA (*f*_MMA_) before the copolymerization reaction and those incorporated in the PSMMA copolymers (*F*_St_) and (*F*_MMA_).

Copolymer PSMMA	*f*_St_	*f*_MMA_	*F*_St_		*F*_MMA_		Conversion (%)
			^13^C NMR	^1^H NMR	^13^C NMR	^1^H NMR	
PSMMA10	0.094	0.905	0.166	0.180	0.834	0.82	8.92
PSMMA25	0.237	0.763	0.578	0.545	0.422	0.455	5.11
PSMMA50	0.444	0.555	–	0.608	–	0.392	6.52
PSMMA75	0.650	0.350	–	0.560	–	0.440	5.42
PSMMA90	0.892	0.107	–	0.95	–	0.05	7.37

### Characterization

The molecular weights of the prepared homopolymers and copolymers were estimated in THF at 30 °C by SEC on a Varian apparatus equipped with a JASCO type 880-PU HPLC pump, refractive index and UV detectors and TSK Gel columns calibrated with polystyrene standards. The FTIR spectra of PSt, PMMA and PSMMA were recorded by a Perkin Elmer 1000 instrument spectrophotometer at room temperature. In all cases, at least 32 scans with an accuracy of 2 cm^−1^ were signal-averaged. The film samples obtained were transparent and sufficiently thin to obey the Beer Lambert law. The structure, microstructure of polymers and the composition in comonomer units (St and MMA) contained in PSMMA samples were determined quantitatively in THF-*d*_8_ by ^1^H and ^13^C NMR spectroscopy at 500 and 200 MHz, respectively. The glass transition temperatures (*T*_g_) of PSt, PMMA homopolymers and PSMMA copolymer films were measured with DSC (Shimadsu DSC 60), previously calibrated with indium. Samples weighing between 10 and 12 mg were packed in aluminum DSC pans before placing in DSC cell. The samples were heated from 20 to 200 °C at a heating rate of 20 °C min^−1^. To reduce the experimental errors due to the instrument measurements and weighing, the results of characterization were taken from the arithmetic average realized for three measurements. The thermal decomposition temperatures of PMMA, PSt and PSMMA samples were determined by a Thermal Analysis SDT2960 Simultaneous DSC-TGA instrument. Measurements were carried out under nitrogen atmosphere. Samples driyed at 60 °C under high vacuum during 24 h were weighing between 10 and 12 mg before scanning in the temperature range 25–500 °C at a heating rate of 10 °C min^−1^. TG-DTG thermograms were recorded simultaneously and the results were traited by TA Instruments Universal Analysis 2000 program. The surface morphologies of pure homopolymers and copolymers were examined by a JEOL JSM 6360LV scanning electron microscope (SEM) at an acceleration voltage of 3.00 and 5.00 kV. Samples were coated with a thin layer of gold to reduce any build-up on the surfaces.

## Results and discussion

### Polymerization and copolymerization of St and MMA

**Scheme 1.**
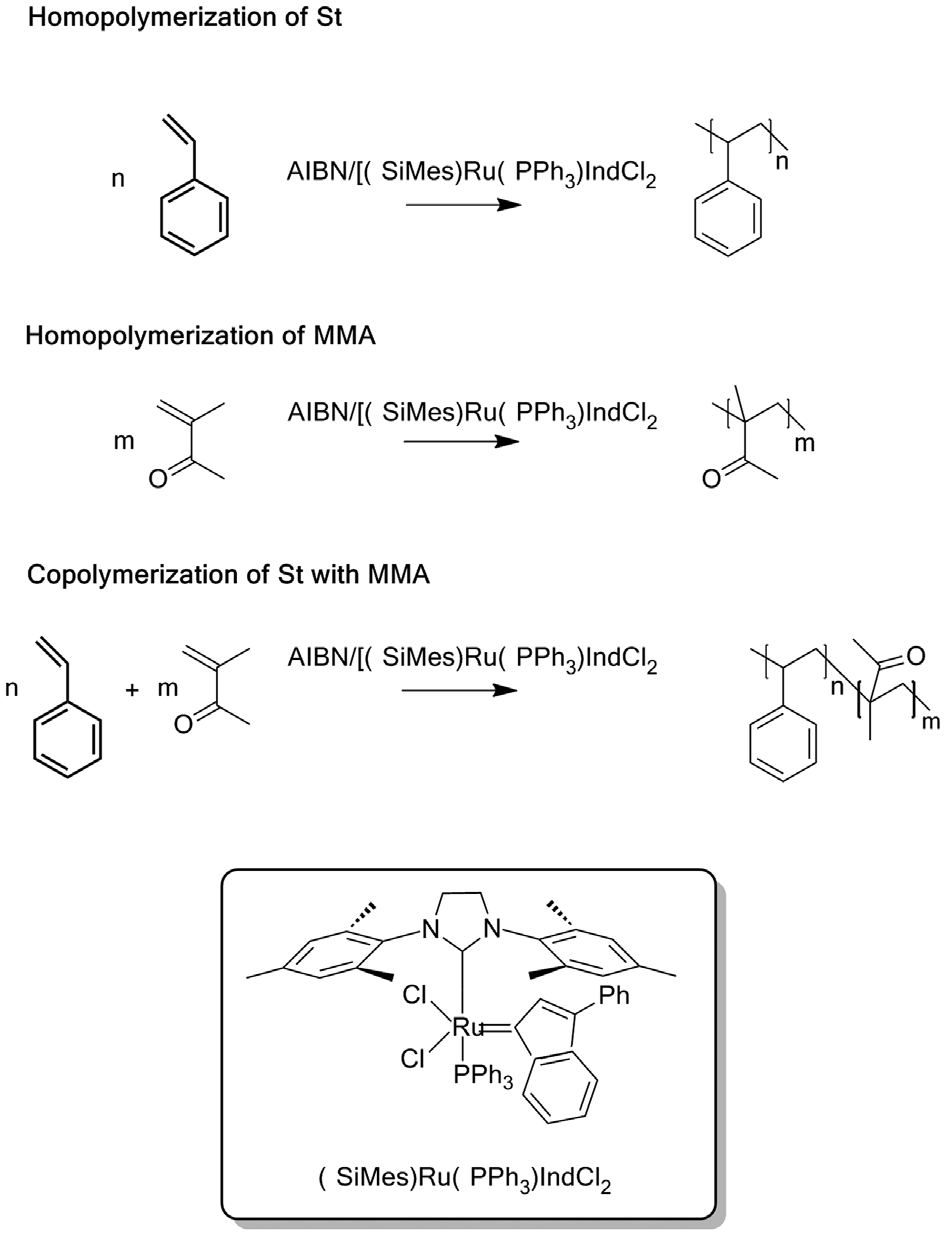
Scheme of the polymerization and copolymerization of styrene and methyl methacrylate using the M_20_/AIBN system.

The polymerization and copolymerization of St and MMA was carried out in bulk and in the presence of the ruthenium complex combined with AIBN as a free-radical generator according to the Scheme 1. It should be noted that in the absence of AIBN, no reaction was observed. AIBN is considered as standard initiator and, when used alone, is known to promote the radical polymerization of vinylic monomers affording high yields of polymers characterized by high molecular weights with large molecular distribution index and random tacticity. The combination of ruthenium dichloride–indenylidene complex with AIBN as showed in Table 1 provided also high yields in respect to the limit of the polymerization’s kinetics. Indeed, in bulk polymerization, the dramatic increase in viscosity with time led to a decrease in the polymerization rate, most notably when larger amounts of starting material were used. This phenomenon was supported by the reproducibility of the observed yield (74–89 wt%) for all reactions after 2 h. Comparable yields and molecular weights were obtained by Rabea and Zhu [25] during 5 h in the polymerization of MMA using the bulk ATRP of methyl methacrylate (MMA) employing an initiator for continuous activator regeneration method.

### Effect of the initiator system aging time

**Scheme 2.**
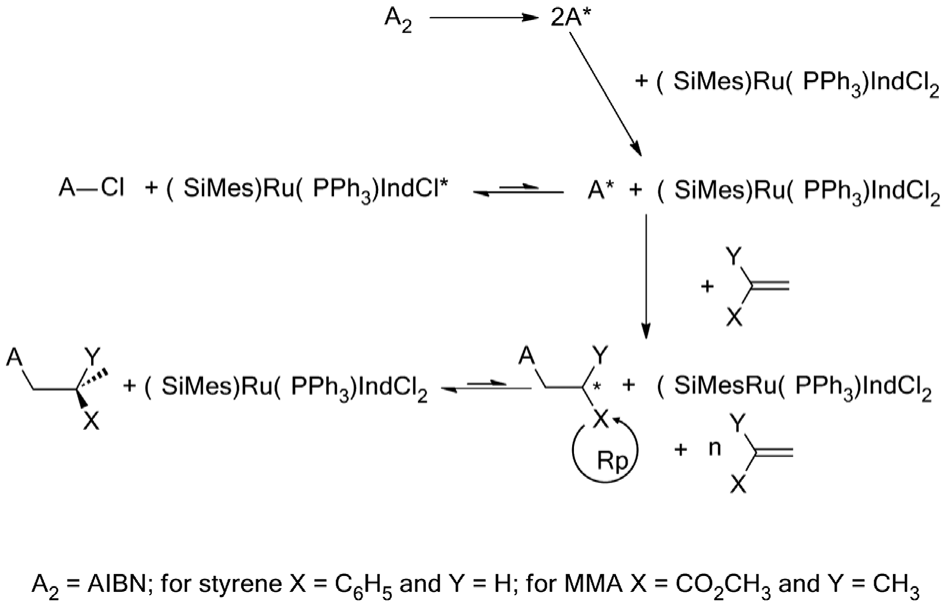
Proposed mechanism of polymerization and copolymerization of styrene and methyl methacrylate using the M_20_/AIBN system.

The effect of the aging time of the initiator system on the polymerization results is examined for PSt and MMA at 80 °C during 5, 25, 60 and 120 min prior addition of monomer and the results obtained are gathered in Tables 3 and 4, respectively. As it can be seen from these data the yield and the average molecular weight for both styrene and methyl methacrylate slowly decrease with increasing the aging time, while the polydispersity index remains practically unchanged. This finding seems to indicate that slow interactions were occurred during the aging time between AIBN and M_20_ before addition the monomer leading to species formation according to Scheme 2.

**Table 3.** Variation of the yield, molecular weight and polydispersity index in the polymerization of PSt vs. the aging time of the initiator system.

Experiment	Aging time (min)	Yield (%)	${\overset{¯}{M}}_{\text{n}}$ (g mol^−1^) × 10^−4^	${\overset{¯}{M}}_{\text{w}}$ (g mol^−1^) × 10^−4^	$\frac{{\overset{¯}{M}}_{\text{w}}}{{\overset{¯}{M}}_{\text{n}}}$
1	5	79.18	9.58	12.17	1.27
2	25	72.26	9.04	11.03	1.22
3	30	65.35	7.52	9.85	1.32
4	60	58.10	7.42	8.89	1.20
5	120	43.26	5.32	6.75	1.27

Notes:

Aging temperature: 80 °C; polymerization temperature: 80 °C; polymerization time: 2 h; St = 3.64 g (3.50 × 10^−2^ mol); AIBN = 2.0 mg (1.22.10^−5^ mol); M_20_/AIBN = 1.0 (mol mol^−1^).

**Table 4.** Variation of the yield, molecular weight and polydispersity index in the polymerization of MMA vs. the aging time of the initiator system.

Experiment	Aging time (min)	Yield (%)	${\overset{¯}{M}}_{\text{n}}$ (g mol^−1^) × 10^−4^	${\overset{¯}{M}}_{\text{w}}$ (g mol^−1^) × 10^−4^	$\frac{{\overset{¯}{M}}_{\text{w}}}{{\overset{¯}{M}}_{\text{n}}}$
1	5	78.23	9.68	11.90	1.23
2	25	59.53	6.97	9.03	1.30
3	30	52.25	5.92	7.91	1.34
4	60	40.90	5.41	6.30	1.16
5	120	38.86	4.95	6.01	1.21

Notes:

Aging temperature: 80 °C; polymerization temperature: 80 °C; polymerization time: 2 h; MMA = 3.74 g (3.74 × 10^−2^ mol); AIBN = 2.0 mg (1.22.10^−5^ mol); M_20_/AIBN = 1.0 (mol mol^−1^).

The species amount resulted in these conditions, which increases with aging time, reduce considerably the catalytic efficiency of AIBN without disrupt the dispersity of the polymer obtained by the initiator system. Indeed, an increase in the aging time from 5 to 120 min decreases about two folds the yield and the molecular weight of the polymer obtained. The decrease in the polymer yield is probably due to a reduction of the transfer reactions and the decrease of the molecular weight of the polymer resulted can be explained by a reduction of the combination reactions in the termination step of the polymerization. This finding allow me to say that the use of the AIBN combined with M_20_ as initiator system can be also used to control the magnitudes of the yield and the molecular weight.

### Structure of homopolymers and copolymers

**Figure 1.**
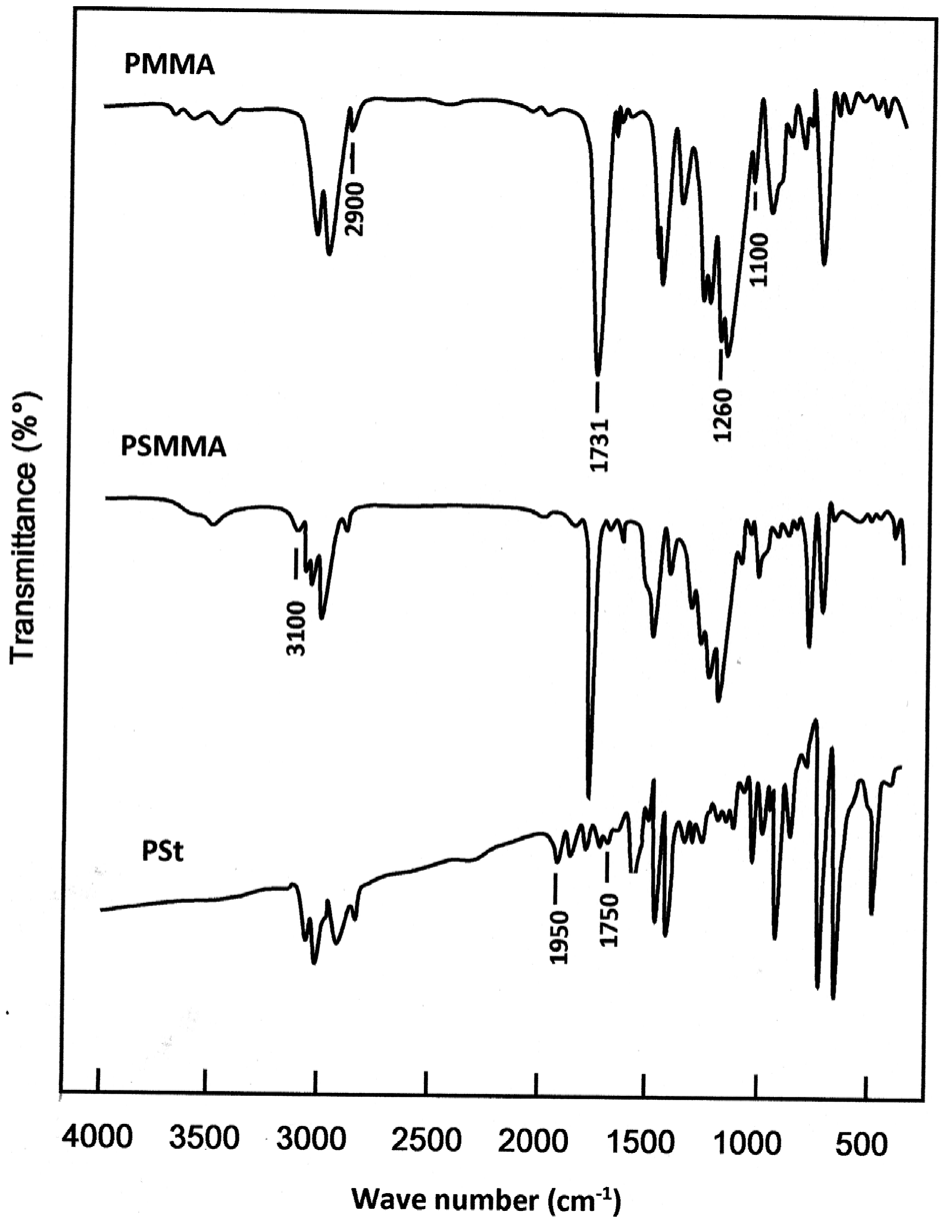
FTIR spectra of PSt, PMMA and PSMAA25 films.

The structure of PSt, PMMA homopolymers and PSMMA copolymers were confirmed by FTIR spectroscopy (Figure 1), through the disappearance of the absorption band of the vinylic group (C=C) of the monomers and the presence of the different absorption bands characterizing the two monomer units. Indeed, as it can be seen from the PSMMA spectrum the MMA units in the copolymer is confirmed by the appearance of the sharp peak at 1731 cm^−1^ assigned to the vibration of the carbonyl group, the wide peak localized between 1000 and 1260 cm^−1^ attributed to the vibration of the stretching of the C–O ester bonds and the less intense bands localized between 2900 and 3100 cm^−1^ [26,27] The styryl unit is confirmed by the presence in the same spectrum by the typical absorption peaks of the phenyl group between 1750 and 1950 cm^−1^.

**Figure 2.**
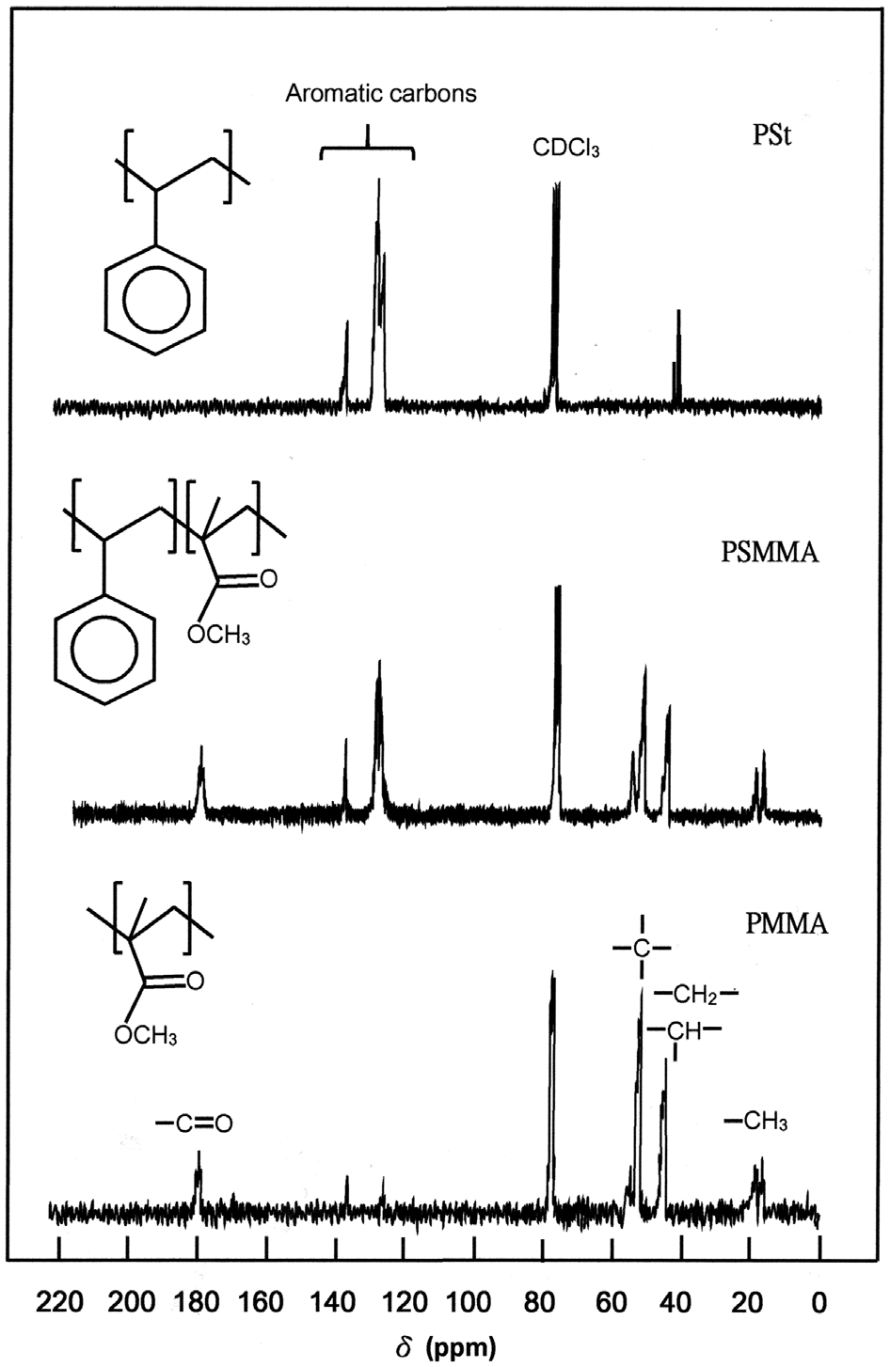
^13^C NMR spectra of PSt, PMMA and PSMMA50 obtained in CDCl_3_.

The determination of the molar fraction of comonomer methyl methacrylic acid, *X*_MMA_ in PSMMA was based on the quantitative comparison of the carbon in α-CH_3_ group of methacrylyl units localized between 17.0 and 20.0 ppm and that of the ethylenyl carbon (–CH_2_–) common to the two different units in the main chain shown in the ^13^C NMR spectrum (Figure 2) using Equation (1).

(1) $$X_{\text{MMA}}=\frac{4\delta_{{\text{CH}}_{3}}}{3\delta_{{\text{CH}}_{2}}}$$

**Figure 3.**
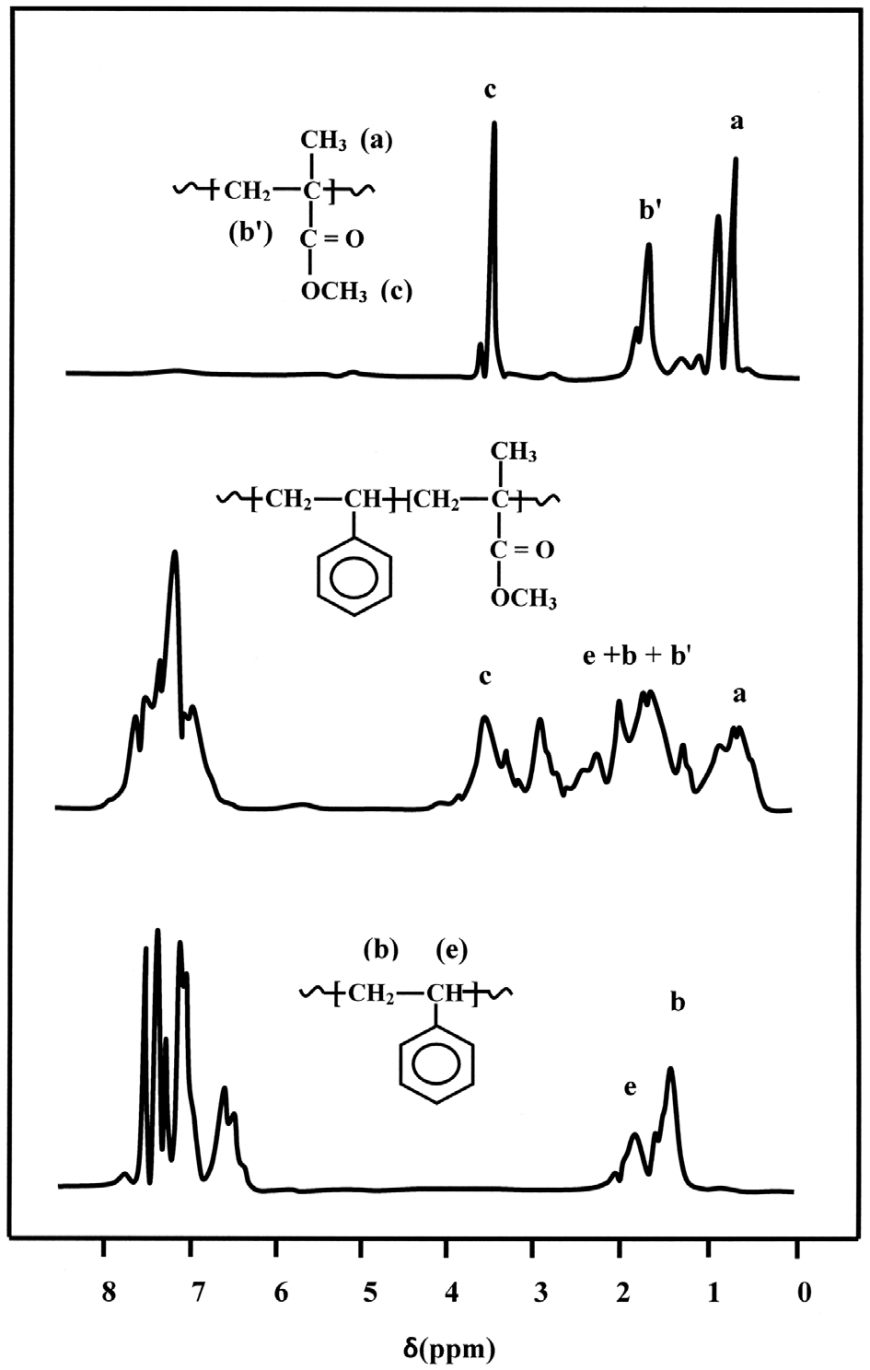
^1^H NMR spectra of PSt, PMMA and PSMMA50 obtained in CDCl_3_.

The molar fraction of MMA in the copolymers was confirmed by ^1^H NMR spectrum (Figure 3) using the surface area of the three protons of methyl group (*a*) localized between 0.50 and 1.20 ppm and that of the five protons of ethynyl group (2*b* + 2*b*′ + *e*) localized between 1.20 and 2.60 ppm using Equation (2).

(2) $$X_{\text{MMA}}=\frac{3\delta (a)}{\delta \left(a\right)+3\delta \left(e+b+b^{\prime }\right)}$$

where *δ*(*a*) and *δ*(*e* + *b* + *b*′) are the surface area of the signal of three protons attributed to the methyl group (*a*) of the MMA and that of the ethynyl protons (*e* + *b* + *b*′), respectively and the results obtained by the two methods are gathered in Table 2.

### Distribution of co-monomeric units in the copolymers

The distribution of St and MMA in the PSMMA chains was determined from the reactivity ratios (*r*_St_ and *r*_MMA_) for the St/MMA copolymerization process. These were calculated from the monomer feed ratios and the copolymer compositions obtained previously by NMR, through the Mayo–Lewis method.[28]

The Mayo–Lewis method is a procedure used to determine the reactivity ratios. Using this method, *r*_St_ values are plotted as a function of various assumed values of *r*_MMA_ according to Equation (3) for each experiment with different feed and copolymer compositions.

(3) $$r_{\text{St}}=f\times \left(\frac{r_{\text{St}}+1}{F}-1\right)$$

where $f=\frac{f_{\text{St}}}{f_{\text{MMA}}}$ and $F=\frac{F_{\text{St}}}{F_{\text{MMA}}}$.

**Figure 4.**
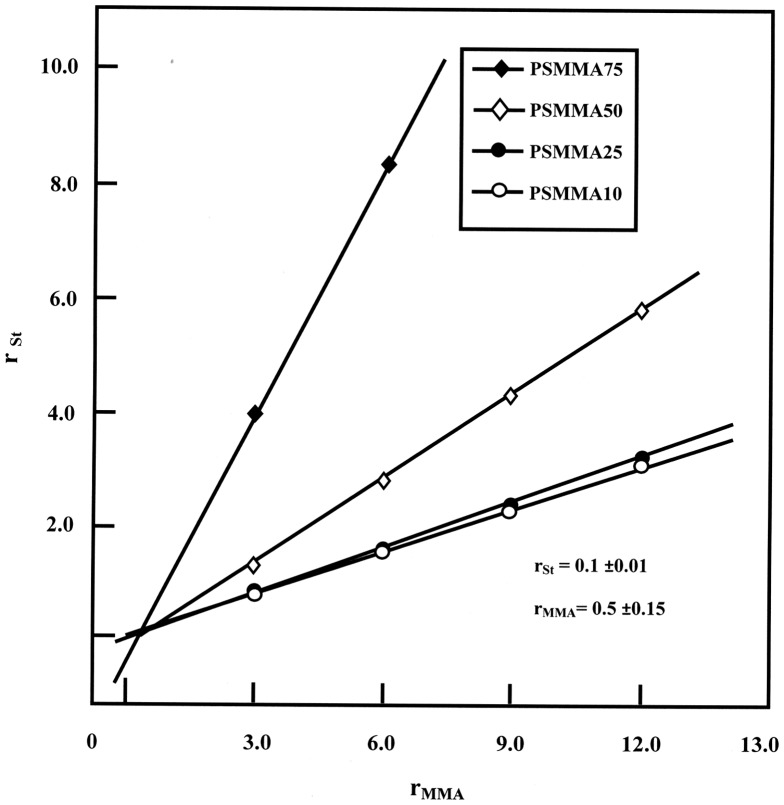
Mayo–Lewis plots, the variation of *r*_St_ vs. *r*_MMA_ for PSMMA.

A straight line was obtained for each experiment and the intersection of the lines led to the best values of *r*_St_ and *r*_MMA_. The obtained Mayo–Lewis plots of *r*_St_ vs. *r*_MMA_ are shown in Figure 4 and the optimal reactivity ratios *r*_St_ and *r*_MMA_ were estimated at 0.10 ± 0.01 and 0.5 ± 0.15, respectively and indicated that an alternating distribution of the co-monomeric units through the PSMMA’s main chain.

### Tacticity of PSt, PMMA and PSMMA

**Figure 5.**
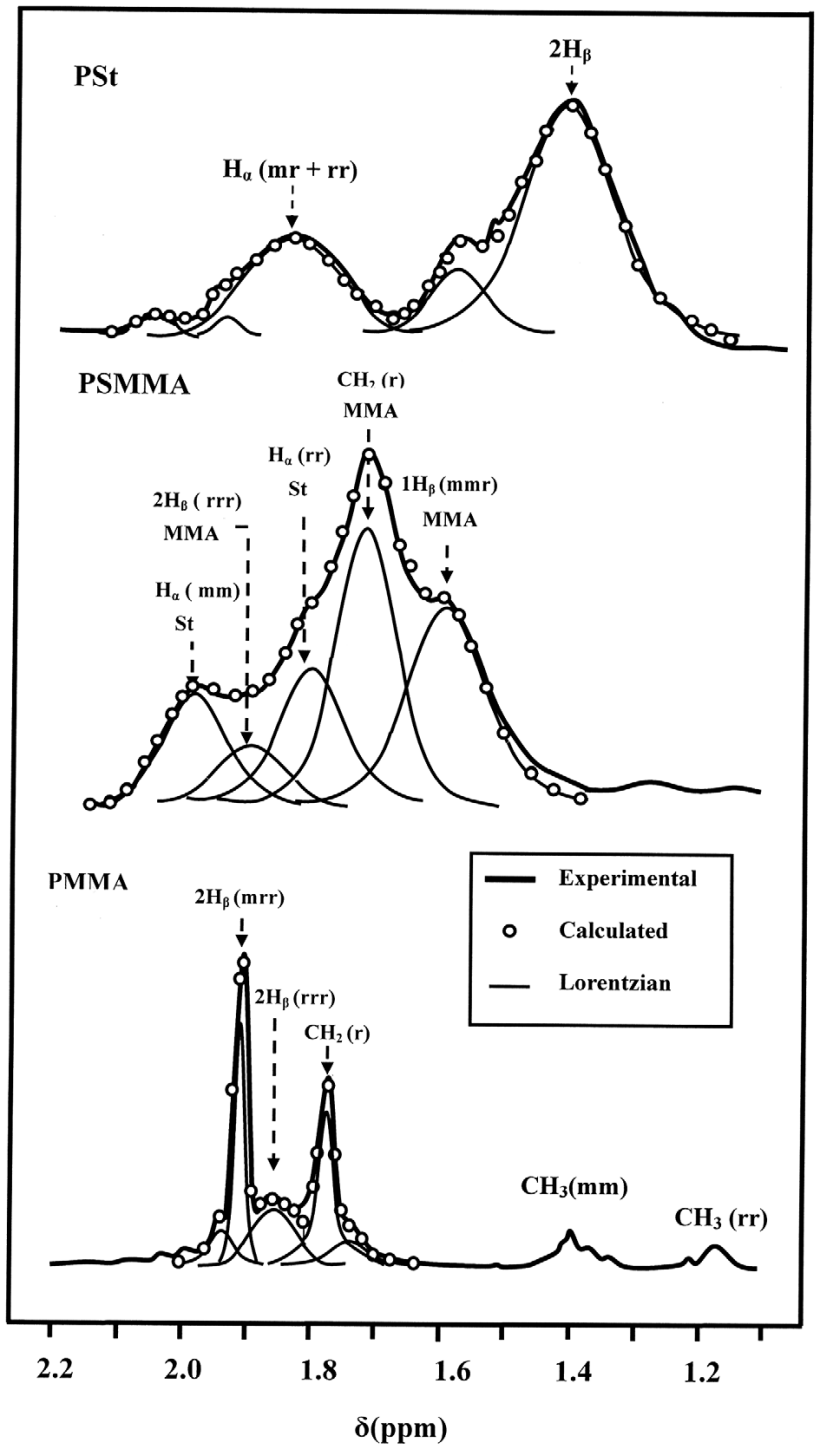
Deconvolution of the ^1^H NMR spectra (1.10–2.15 ppm) of PSt, PSMMA and PSMMA25 via lorentzian peaks.

The tacticity of PSt was determined by ^1^H NMR spectroscopy based on the chemical shift of the H_α_ in PSt (aliphatic CH, 1.10–2.15 ppm) and that of the three protons in PMMA (*α*-methyl group, 1.62–2.07 ppm). The deconvolution of these broad signals of polystyrene and PMMA via lorenzian peaks, as shown in Figure 5, revealed several peaks in this range. The PSt presented two main peaks localized at 1.42 and 1.83 ppm attributed to the 2H_β_ and H_α_ (mr + rr) of the atactic triads respectively, while that of PMMA showed three main peaks at 1.77, 1.85 and 1.91 ppm assigned to the 2H_β_ (mrr), 2H_β_(rrr) tetrads and CH_2_(r) dyads.[29] From the surface area of each absorption peak it was possible to evaluate easily the tacticity of each polymer and copolymers. These results revealed that the PSt presented an isotactic structure at 55%, while that of PMMA, based on the 2H_β_ protons, indicated 72.3% of a syndiotactic structure. Concerning the PSMMA, its geometric structure was essentially atactic.

### Dependence of the molecular weight and molecular weight distribution on the monomer conversion

The average molecular weight of PSt, PMMA and PSMMA with different St/MMA compositions determined by the GPC method were gathered in Table 5. The data showed that the polymers and copolymers synthesized by the ruthenium complex M_20_ and AIBN system led to relatively high polymers (4.33.10^4^–11.721.0^4^) g mol^−1^ with a narrow molecular distribution, in which the polydispersity index varied between 1.10 and 1.40. These results can be easily confirmed by a simple calculation based on the yield of polymerization. For an isomolecular system the molecular weights (*M*_cal_) can be determined directly using Equation (4).

(4) $$M_{\text{cal}}=\frac{N_{\text{M}}\times \text{Yield}\times M_{\text{M}}}{100\times N_{\text{FR}}}$$

**Table 5.** Molecular weight distribution of polystyrene, poly(methylmethacrylate) and poly(styrene-*co*-methylmethacrylate) copolymers with different St/MMA compositions.

System	Composition St:MMA (g%)	${\overset{¯}{M}}_{\text{n}}\times 10^{-4}$ (g mol^−1^)	${\overset{¯}{M}}_{\text{w}}\times 10^{-4}$ (g mol^−1^)	${\overset{¯}{M}}_{\text{cal}}\times 10^{-4}$ (g mol^−1^)	$\frac{{\overset{¯}{M}}_{\text{w}}}{{\overset{¯}{M}}_{\text{n}}}$
PS	–	8.75	11.11	11.84	1.269
PSMMA1	90:10	5.72	6.87	–	1.200
PSMMA2	75:25	5.21	6.50	–	1.247
PSMMA3	50:50	5.97	7.17	–	1.201
PSMMA4	25:75	8.80	9.64	–	1.095
PSMMA5	90:10	4.34	6.10	–	1.407
PMMA	–	11.72	13.33	11.78	1.137

where *N*_M_, *N*_FR_ and *M*_M_ are the mole number of monomer, free radical resulted from the dissociation of initiator and the molecular mass of the monomer, respectively. As it can be seen from the comparison of the molecular weights calculated with those measured (Table 5) reveals very close values, thus confirming the polymerization results, notably the monodispercity character of the polymers synthesized.

### The thermal property analysis

**Figure 6.**
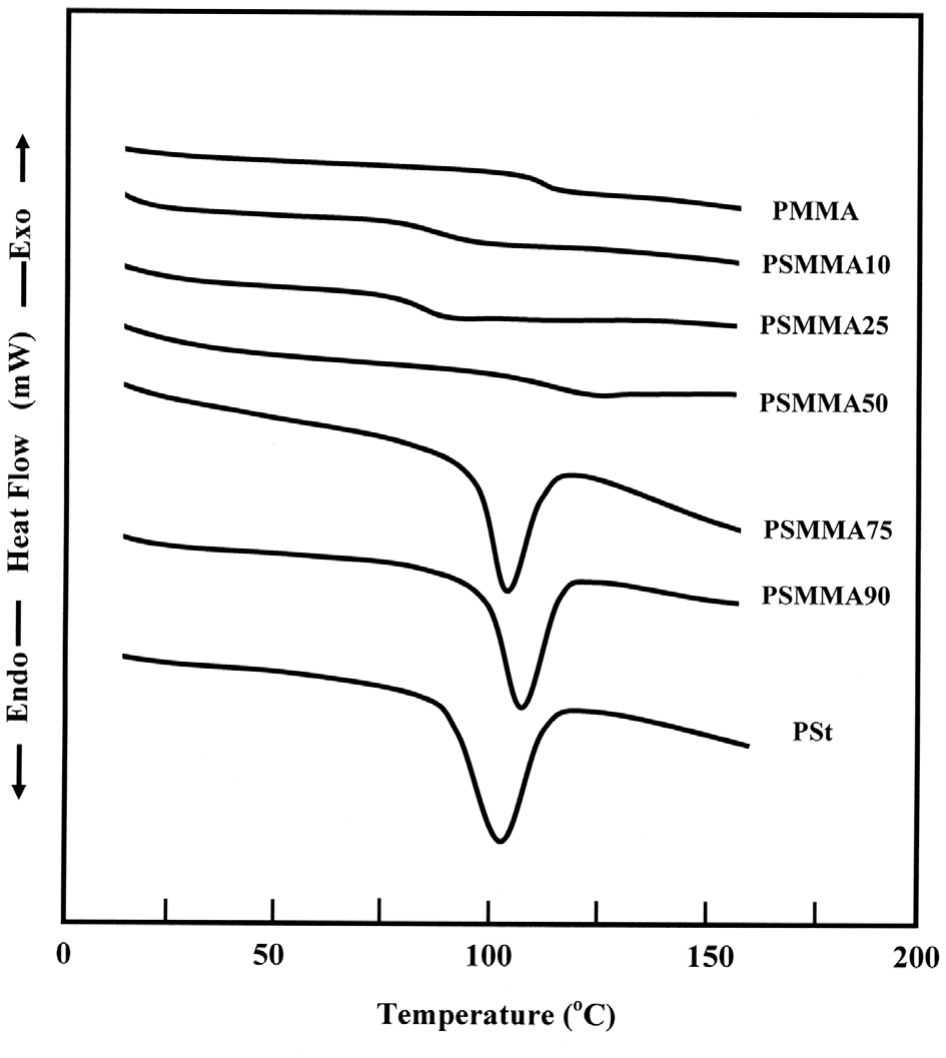
DSC thermograms of PSt, PMMA and PSMAA obtained with a heating rate of 20 °C min^−1^.

DSC thermograms performed on the PSt, PMMA and PSMMA copolymers, in heating mode, showed only a single glass transition temperature, *T*_g_, for all PSMMA samples between those of pure PSt and PMMA, thus suggesting that the copolymerization products were true copolymers and not a blend of two homopolymers. According to the literature, the mixture of PSt with PMMA is immiscible in all ratios,[30-33] and conequently their thermograms revealed two glass transition temperatures attributed to the two homopolymers (Figure 6).

**Figure 7.**
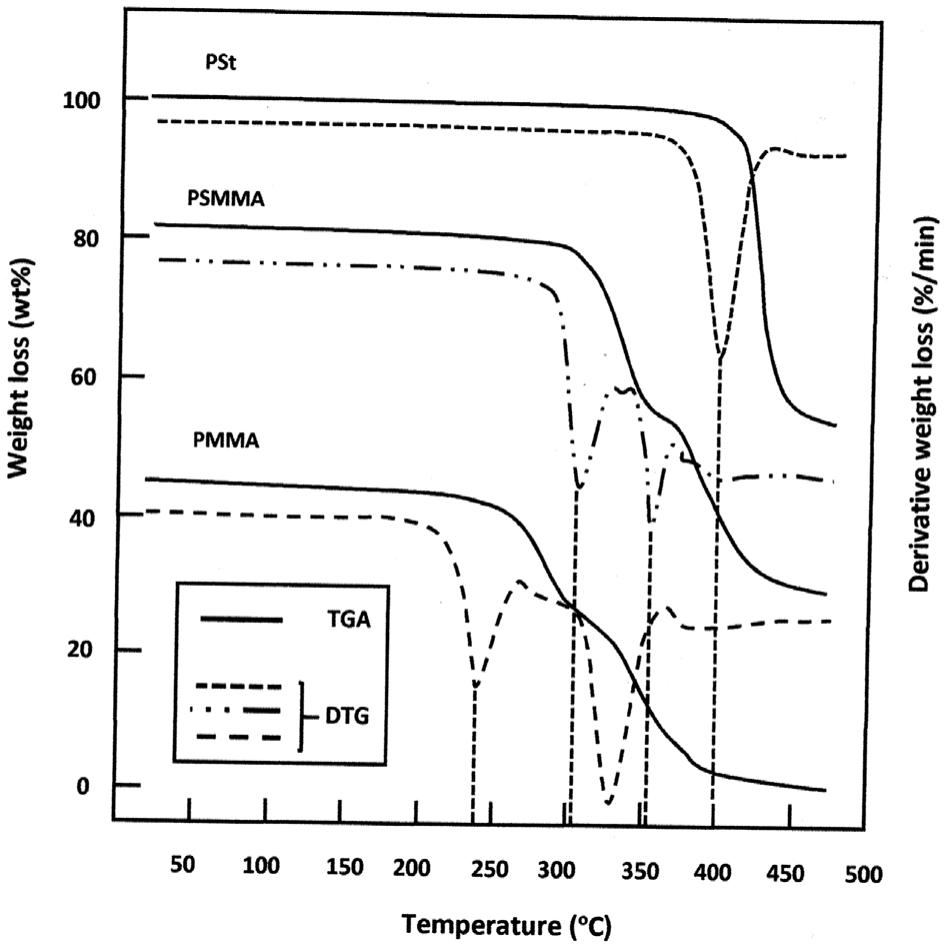
TGA and DTA curves of PMMA, PSt and PSMMA spectra obtained at a heating rate of 10 °C min^−1^.

The TGA method is used in this work to study the thermal stability of the polymers and copolymers synthesized by the M20/AIBN system. Indeed, as it can be observed on the thermograms of Figure 7, the PMMA shows two decomposition stages. The first one which starts at 237 ± 2 °C is attributed to the radical transfer to unsaturated chain ends causing the breakdown of C–C bonds. That at 327 ± 2 °C is associated with random scission of the polymer backbone. These values agree those of the literature.[26,34,35] The thermogram of the pure PSt reveals only one decomposition stage that characterize this polymer in which the weight loss starts at 400 ± 2 °C. This results agree with those of PSt obtained by Hassan et al. [26]. According to Richards and Salter [36]. two processes intervene in the decomposition mechanism of this polymer, (i) weak bond scission and (ii) intermolecular chain transfer. This would support the hypothesis of an activated intermediate. The principal volatile products resulted from the decomposition of polystyrene evolved styrene and toluene and α-methyl styrene. Concerning the PSMMA thermogram two decomposition stages are showed and reveals important shifts in the decomposition temperatures of this copolymer, the first one which begin at 306 ± 2 °C is probably attributed to the radical transfer to unsaturated chain ends for both MMA and Styrene units and the second one at 353 ± 2 °C is attributed to the weak bond scission and intermolecular transfer.

**Figure 8.**
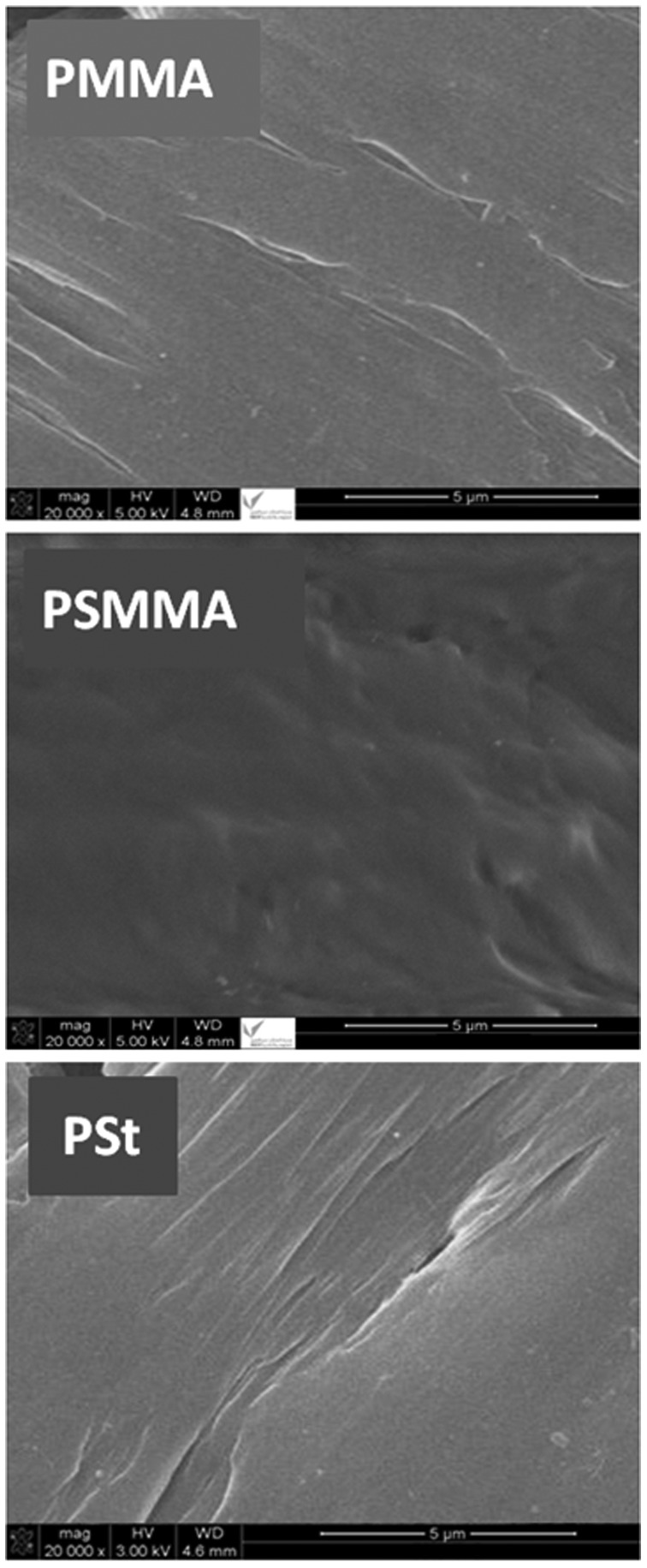
SEM micrographs of PMMA, PSt, and PSMMA specimens.

The surface morphologies of pure PMMA, PSt and PSMMA films obtained by the M20/AIBN combination are showed in Figure 8. As it can be observe from the micrographs of PMMA and PSt films a smooth, uniform and monophasic surface morphologies. Similar surface is also observed for the PSMMA specimen which is devoid of any stress could be reflecting the repulsion forces causing the immiscibility of a pair of polymers. Knowing that the PMMA/PSt blend is immiscible and basing on the criterion that stipulates that an immiscible blend is characterized by a heterogenic surface morphology indicating the presence of stress resulting from the contraction of each constituent due to the electrostatic repulsion forces.[37-40] This clearly says that the different monomer units is really incorporated within the same chain and not in two different chains thus confirming the structure of copolymer obtained.

## Conclusion

The homopolymerization of styrene and methyl methacrylate and their copolymers were successfully performed using the combination of (SiMes)Ru(PPh_3_)IndCl_2_ and AIBN as the catalyst system, at a moderate temperature (80 °C). The efficiency of this catalyst system was demonstrated by obtaining yields compared to that obtained by AIBN alone in the same conditions. The molecular weight distribution performed on PSt, PMMA and the copolymers revealed moderate molecular weights with 8.73.10^4^ and 11.11.10^4^ g mol^−1^and a narrow molecular weight distribution, in which the polydispersity index was 1.13 and 1.26 for PSt and PMMA, respectively. An increase in the aging time from 5 to 120 min decreases about two folds the yield and the molecular weight of the PMMA obtained without disturb its dispersity. The polydispersity index varied between 1.20 and 1.40 for PSMMA, depending on the composition of the comonomer units in the copolymer. The comparison of these results with those obtained through a free radical polymerization, using AIBN alone under the same conditions, indicated clearly that the combination of AIBN with the ruthenium dicloride-indenylidene complex reduced considerably the transfer reactions. The distribution of St and MMA comonomer units in the copolymer chains was analyzed by the Mayo–Lewis method and showed an alternating distribution. The PSMMA copolymers with different St ratios were determined to be essentially atactic. The geometric structures of PSt, PMMA and PSMMA, obtained with this catalytic system, were determined to be respectively 55% of atactic, 72.3% of syndiotactic and majoritarly atactic. It should be noted that the DSC and SEM analysis of PSMMA material confirmed the production of copolymers and not a mixture of two polymers by the apparition of a single *T*_g_ on the thermogram and presents in its micrograph a smooth, uniform and monophasic surface morphologies devoids of any stress could be reflecting the immiscibility if the sample contains a blend of polymers. Finally, by reducing transfer reactions, the system used here led to the CRP.

## Disclosure statement

No potential conflict of interest was reported by the authors.

## Funding

This work was supported by the King Saud University.

## References

[1] Matyjaszewski K, Xia J Atom transfer radical polymerization. Chem. Rev. 2001;101:2921–2990. 10.1021/cr940534g 11749397

[2] Smirnov BR Reversible inhibition of radical polymerization. Vysokomol. Soedin. 1990;32:583–589 [in Russian].

[3] Smirnov BR, Plotnikov VD, Ozerkovskii BV, et al. Catalysis of chain transfer and oligometer structure in radical polymerization of styrene in the presence of cobalt complexes of porphyrins. Polym. Sci. U.S.S.R. 1981;23:2807–2816. 10.1016/0032-3950(81)90056-3

[4] Smirnov BR, Belgovskii IM, Ponomarev GV, et al. Catalysis of the reaction of chain transfer onto a monomer in the radical polymerization. Dokl. Akad. Nauk. SSSR. 1980;254:127–130.

[5] Islamova MR, Nazarova SV, Koifman OI Porphyrins and their metal complexes in radical polymerization of vinyl monomers. Макрогетероциклы/Macroheterocycles. 2011;4:97–105. 10.6060/mhc2011.2.06

[6] Koifman OI, Ageeva TA Porfirinpolimery [Porphyrinpolymers]. Moskva: Izd Fiz-mat Liter; 2006; 195 p. [in Russian].

[7] Lu G, Li Yi-Min, Lu Chun-Hua, et al. AGET ATRP of methyl methacrylate by silica-gel-supported copper(II) chloride/2-(8-heptadecenyl)-4,5-dihydro-1H-imidazole-1-ethylamine. Des. Monomers Polym. 2010;13:509–522. 10.1163/138577210X530585

[8] Kumar M, Kannan T Dimethylaminoethyl methacrylate functionalized montmorillonite for the preparation of polymer-montmorillonite nanocomposites through iniferter-based controlled radical polymerization of methyl methacrylate and styrene. Polym.-Plast. Technol. Eng. 2014;53:604–612. 10.1080/03602559.2013.854390

[9] Okcu SS, Durmaz YY, Yagci Y Synthesis and characterization of telechelic block co-polymers by combination of atom transfer radical polymerization and click chemistry processes. Des. Monomers Polym. 2010;13:459–472.

[10] Cankaya G, Bicak N Zinc powder-alkyl halide: a radical initiation system for living/controlled polymerization of vinyl monomers. Des. Monomers Polym. 2015;18:27–34. 10.1080/15685551.2014.947550

[11] Wayland BB, Poszmik G, Mukerjee SL Living radical polymerization of acrylates by organocobalt porphyrin complexes. J. Am. Chem. Soc. 1994;116:7943–7944. 10.1021/ja00096a080

[12] Lu Z, Fryd M, Wayland BB New life for living radical polymerization mediated by cobalt(II) metalloradicals. Macromolecules. 2004;37:2686–2687. 10.1021/ma035924w

[13] Wayland BB, Poszmik G, Fryd M Metalloradical reactions of rhodium (II) porphyrins with acrylates: reduction, coupling, and photopromoted polymerization. Organometallics. 1992;11:3534–3542. 10.1021/om00059a016

[14] Kuroki M, Aida T, Inoue S Novel photoinduced carbon–carbon bond formation via metal-alkyl and -enolate porphyrins-visible light-mediated polymerization of alkyl methacrylate catalyzed by aluminum porphyrin. J. Am. Chem. Soc. 1987;109:4737–4738. 10.1021/ja00249a056

[15] Hosokawa Y, Kuroki M, Aida T, et al. Controlled synthesis of poly(acrylic esters) by aluminum porphyrin initiators. Macromolecules. 1991;24:824–829. 10.1021/ma00004a002

[16] Dragutan V, Dragutan I, Verpoort F Ruthenium indenylidene complexes. Platinum Met. Rev. 2005;49:33–40. 10.1595/147106705X24580

[17] De Frémont P, Clavier H, Montembault V, et al. Ruthenium–indenylidene complexes in ring opening metathesis polymerization (ROMP) reactions. J. Mol. Catal. A: Chem. 2008;283:108–113. 10.1016/j.molcata.2007.11.038

[18] Monsaert S, De Canck E, Drozdzak R, Van Der Voort P, Hendrickx PMS, Martins JC, Verpoort F Ruthenium–indenylidene complexes bearing saturated N-heterocyclic carbenes: synthesis and application in ring-closing metathesis reactions In Dragutan V, Demonceau A., Dragutan I., & , Finkelshtein E.S., editors. Green metathesis chemistry: great challenges in synthesis, catalysis and nanotechnology. Springer Science + Business Media B.V. 2010. Green metathesis chemistry, NATO science for peace and security series A: chemistry and biology, Belgium; 2010 p. 31–38.

[19] Jafarpour L, Schanz HJ, Stevens ED, et al. Indenylidene−imidazolylidene complexes of ruthenium as ring-closing metathesis catalysts. Organometallics. 1999;18:5416–5419. 10.1021/om990587u

[20] Opstal T, Verpoort F From atom transfer radical addition to atom transfer radical polymerisation of vinyl monomers mediated by ruthenium indenylidene complexes. New J. Chem. 2003;27:257–262. 10.1039/b210040a 12833346

[21] Simal F, Sebille S, Hallet L, et al Evaluation of ruthenium-based catalytic systems for the controlled atom transfer radical polymerisation of vinyl monomers. Macromol. Symp. 2000;161:73–86. 10.1002/(ISSN)1521-3900

[22] De Clercq B, Verpoort F Atom transfer radical polymerization of vinyl monomers mediated by a new class of neutral and cationic ruthenium alkylidene catalysts containing a 1,3-dimesityl-4,5-dihydroimidazol-2-ylidene and a Schiff base ligand. Polym. Bull. 2003;50:153–160. 10.1007/s00289-003-0149-9

[23] Diaz MOG, Morales SL, Le Lagadec R, et al Homogeneous radical polymerization of 2-hydroxyethyl methacrylate mediated by cyclometalated cationic ruthenium(II) complexes with PF_6_ ^−^ and Cl^−^ in protic media. J. Polym. Sci. Part A: Polym. Chem. 2011;49:4562–4577. 10.1002/pola.v49.21

[24] Urbina-Blanco CA, Manzini S, Gomes JP, et al Simple synthetic routes to ruthenium–indenylidene olefin metathesis catalysts. Chem. Commun. 2011;47:5022–5024. 10.1039/c1cc10741k 21423960

[25] Rabea AM, Zhu S Pushing monomer conversions high in bulk ATRP: the effects of ICAR agent concentrations on the system livingness and polymer molecular weight control In Matyjaszewski K, Sumerlin BS, Tsarevsky NV, Chiefari J, editors. Controlled radical polymerization: mechanisms, Chapter 9, Vol. 1187, p. 159–169. Washington, DC: ACS Symposium Series May 2015. doi:10.1021/bk-2015-1187.ch009.

[26] Hassan M, Reddy KR, Haque E, et al J. Colloid Interface Sci. 2013;410:43–51. 10.1016/j.jcis.2013.08.006 24034217

[27] Jaymand M Synthesis and characterization of syndiotactic polystyrene-graft-poly(methacrylate) via free-radical polymerization. Polym.-Plast. Technol. Eng. 2012;51:514–520. 10.1080/03602559.2012.654574

[28] Mayo FR, Lewis FM Copolymerization. I. A basis for comparing the behavior of monomers in copolymerization; the copolymerization of styrene and methyl methacrylate. J. Am. Chem. Soc. 1944;66:1594–1601. 10.1021/ja01237a052

[29] Pham QT, Petiaud R, Waton H, Liauro-Darricades MF Proton and carbon NMR spectra of polymers. Bocca Raton, FL: CRC Press; 1991.

[30] Matyjaszewski K The importance of exchange reactions in controlled/living radical polymerization in the presence of alkoxyamines and transition metals. Macromol. Symp. 1996;111:47–61. 10.1002/masy.v111.1

[31] Kaniappan K, Latha S Certain investigations on the formulation and characterization of polystyrene/poly (methyl methacrylate) blends. Int. J. ChemTech. Res. 2011;3:708–717.

[32] Tanaka K, Takahara A, Kajiyama T Film thickness dependence of the surface structure of immiscible polystyrene/poly(methyl methacrylate) blends. Macromolecules. 1996;29:3232–3239. 10.1021/ma951140+

[33] MacCallum JR The thermal degradation of poly(methyl methacrylate). Makromol. Chem. 1965;83:137–147. 10.1002/macp.1965.020830111

[34] Mu D, Li JQ, Zhou YH Modeling and analysis of the compatibility of polystyrene/poly(methyl methacrylate) blends with four inducing effects. J. Mol. Model. 2011;17:607–619. 10.1007/s00894-010-0755-z 20524022

[35] Lee LH, Chen WC High-refractive-index thin films prepared from trialkoxysilane-capped poly(methyl methacrylate)-titania materials. Chem. Mater. 2001;13:1137–1142. 10.1021/cm000937z

[36] Richards DH, Salter DM, Thermal degradation of vinyl polymers III-A radiochemical study of intermolecular chain transfer in the thermal degradation of polystyrene. Polymer. 1967;8:153–159. doi:10.1016/0032-3861(67)90019-5.

[37] Morin C, Ikeura-Sekiguchi H, Tyliszczak T, et al X-ray spectromicroscopy of immiscible polymer blends: polystyrene-poly(methyl methacrylate). J. Electron Spectrosc. Relat. Phenom. 2001;121:203–224. 10.1016/S0368-2048(01)00335-8

[38] Li X, Han Y, An L Surface morphology control of immiscible polymer-blend thin films. Polymer. 2003;44:8155–8165. 10.1016/j.polymer.2003.10.012

[39] Luo WH, Zhou NQ, Peng XF Rheological behavior of immiscible PS/PMMA blends during dynamic plasticating extrusion. Int. Polym. Process. 2008;23:317–322. 10.3139/217.2142

[40] Mathur V, Sharma K Thermal response of polystyrene/poly methyl methacrylate (PS/PMMA) polymeric blends. Heat Mass Transfer. 2016;1–11. doi:10.1007/s00231-016-1779-4.

